# Elements of effective community engagement: lessons from a targeted malaria elimination study in Lao PDR (Laos)

**DOI:** 10.1080/16549716.2017.1366136

**Published:** 2017-09-15

**Authors:** Bipin Adhikari, Christopher Pell, Koukeo Phommasone, Xayaphone Soundala, Palingnaphone Kommarasy, Tiengkham Pongvongsa, Gisela Henriques, Nicholas P. J. Day, Mayfong Mayxay, Phaik Yeong Cheah

**Affiliations:** ^a^ Mahidol-Oxford Tropical Medicine Research Unit, Faculty of Tropical Medicine, Mahidol University, Bangkok, Thailand; ^b^ Centre for Tropical Medicine and Global Health, Nuffield Department of Medicine, Churchill Hospital, Oxford, UK; ^c^ Kellogg College, University of Oxford, Oxford, UK; ^d^ Centre for Social Science and Global Health, University of Amsterdam, Amsterdam, The Netherlands; ^e^ Amsterdam Institute for Global Health and Development, Amsterdam, The Netherlands; ^f^ Lao-Oxford-Mahosot Hospital-Wellcome Trust Research Unit (LOMWRU), Microbiology Laboratory, Vientiane, Laos; ^g^ Savannakhet Provincial Health Department, Savannakhet Province, Laos; ^h^ Faculty of Postgraduate Studies, University of Health Sciences, Vientiane, Laos

**Keywords:** Community, community engagement, Laos, malaria, elimination, mass drug administrations

## Abstract

**Background**: Mass drug (antimalarial) administration (MDA) is currently under study in Southeast Asia as part of a package of interventions referred to as targeted malaria elimination (TME). This intervention relies on effective community engagement that promotes uptake and adherence in target communities (above 80%).

**Objective**: Based on the experienced of designing and implementing the community engagement for TME in Laos, in this article we aim to present the elements of effective community engagement for mass antimalarial administration.

**Methods**: The design and implementation of community engagement, which took place from September 2015 to August 2016 was recorded as field notes, meeting minutes and photographs. These data underwent qualitative content analysis.

**Results**: The community engagement strategy that accompanied TME in Laos was successful in terms of contributing to high levels of participation in mass anti-malarial administration (above 85%). Based on the experience of designing and implementing the community engagement, five key elements were identified: (1) stakeholder and authority engagement, which proceeded from national level, to regional/district and local level; (2) local human resources, particularly the recruitment of local volunteers who were integral to the design and implementation of activities in the study villages; (3) formative research, to rapidly gain insight into the local social and economic context; (4) responsiveness whereby the approach was adapted according to the needs of the community and their responses to the various study components; and (5) sharing control/leadership with the community in terms of decisions on the organization of TME activities.

**Conclusions**: The community engagement that accompanied TME in Laos had to deal with challenges of implementing a complex study in remote and linguistically isolated villages. Despite these challenges, the study recorded high population coverage. Lessons learnt from this experience are useful for studies and intervention programs in diverse contexts.

## Background

Although global malaria-related morbidity and mortality is declining, artemisinin-resistant *Plasmodium falciparum* parasites have recently been detected in South East Asia. The possibility of these parasites spreading to Africa – a potential public health disaster – has prompted a shift from malaria control to elimination in South East Asia [1–4]. As part of a package of interventions, referred to as Targeted Malaria Elimination (TME), mass antimalarial administration – the presumptive treatment of an entire community to interrupt completely local malaria transmission – is currently under study across the region [5].

The effectiveness of mass antimalarial administration is predicated on several factors, including high population coverage [6]. Achieving high coverage is a challenge for several reasons. For example, explaining the rationale for taking antimalarials when asymptomatic can be difficult because of the complex scientific concepts that underlie the approach [7]. Furthermore, target communities are often remote with poor access, villagers tend to be highly mobile and populations can be divided by political affiliations [8–12].

To promote coverage, programs of mass drug administration (MDA) often incorporate community engagement [5–7,11,13]. Most scientific reports of mass antimalarial administration however offer little detail on how community engagement is designed and what activities it entails [11]. Community engagement is often simply short-hand for activities, such as discussions with village leaders and mass meetings to provide health education [11]. Although such activities are likely to have limited impact in terms of uniting fragmented communities, such activities, if appropriately designed can, for example, promote understanding of the benefits of MDA and contribute to uptake in communities [9].

In the wider global health literature, community engagement has multiple meanings. Some scholars view it as an essential component of ethical good practice in research and health programs; others prioritize community health benefits through ensuring the success of an intervention [14]. The divergent definitions and goals of community engagement complicate the evaluation and comparison of community engagement across studies [14]. Efforts have been made to identify common elements in ‘successful’ community engagement, with some scholars providing general principles for broad health-related research [15] and others building on experience from specific research on vector-borne diseases [16].

Laos, along with other states in the Greater Mekong Sub Region, has committed to the goal of eliminating malaria by 2030 [17,18]. To achieve this, an intensification of malaria prevention and control activities – such as MDA – are required [5]. Understanding effective community engagement to accompany such efforts is imperative to ensure their uptake in the target communities.

From the outset of TME in Laos, a broad definition of community engagement, as a process of working collaboratively with relevant partners who share common goals and interests, was used [11,14,15,19]. Established frameworks [16,19] were used to guide community engagement with the primary aim of promoting uptake of MDA to maximize the probability of interrupting local malaria transmission (which was the overall aim of TME). In target communities, the coverage of TME was very high (above 85%) and, in this article, elements of effective community engagement are identified from the experience of implementing TME in Laos.

### Setting

**Figure 1.**
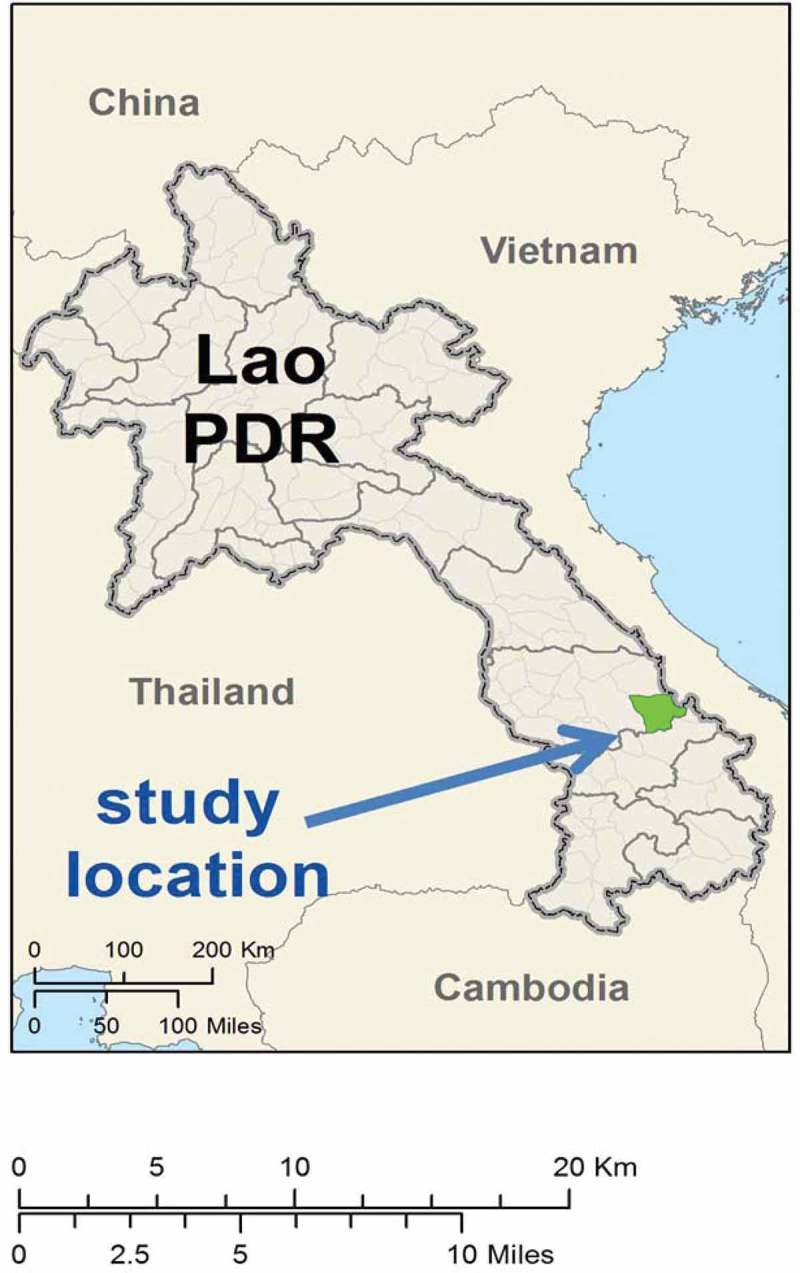
TME study sites in Nong District, Savannakhet province of Laos.

TME was conducted in four randomly selected villages in Nong District, Savannakhet Province, southern Laos (Figure 1). This rural area borders Vietnam and is around five hours drive from the provincial capital, Savannakhet.

Based on the baseline census, over 95% of the residents in the four study villages are from the *Lao Theung* ethnic groups. In the villages – Oi Tan Tip (OTP; population: 512), Phoun Mak Mee (PMM; population: 480), Thate (TT; population: 526), and Xuang Tai (XT; population: 371) – people mostly depend on subsistence agriculture, mainly swidden farming. There are many Vietnamese-owned rubber plantations where native Laotians work to generate cash. In emergencies, almost all villagers can access cash through (rearing and) selling domestic animals, mainly chickens, pigs, cows, goats, and buffaloes.

Only one village (PMM) has a health center within the confines of the settlement. Residents of the other villages travel up to 10km to reach the nearest health facility. Roads are also often impassable after heavy rains and there is no public transport available. A few village heads and village leaders own small hand tractors and few villagers have motorbikes.

Each village is governed by the village head. Other elements of village hierarchies included ‘senior’ (*nayhom)*, security head, youth leaders, women union head, Lao trade union head, and village health volunteers. Villages are divided into units, each with a unit head. Three TME villages contain sub-villages and each sub-village has its own hierarchy. Although the main village head can exert influence over sub-villages, in practice, sub-village heads are ‘in charge’.

During the TME study, no other organizations were implementing research or health interventions. Notably, there was no malaria control program.

### TME in Laos

**Figure 2.**
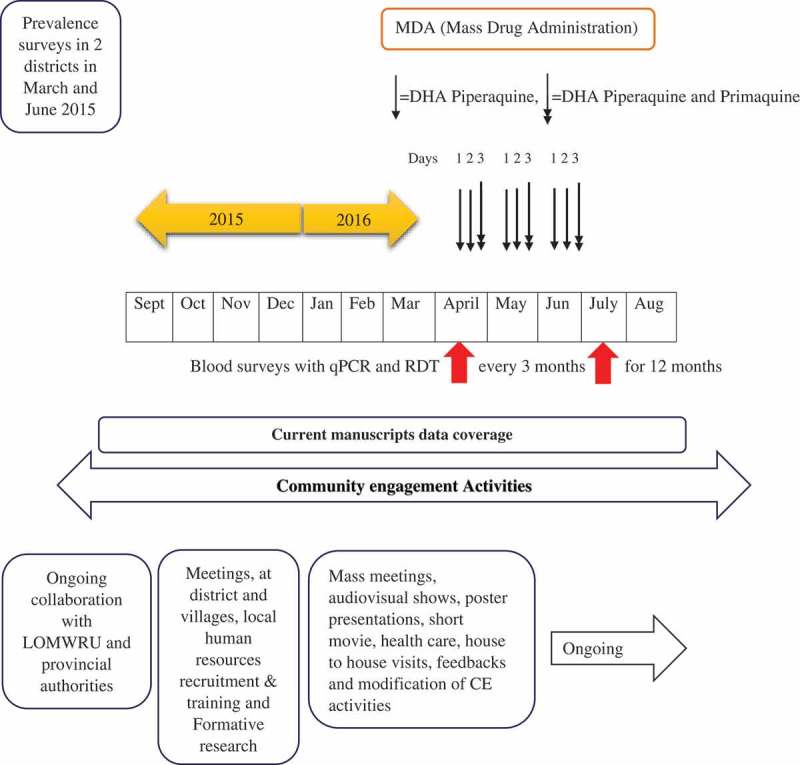
Schematic representation of TME and CE activities.

Four TME villages (two ‘intervention’ and two ‘control’) were selected out of 18 prescreened villages in two district of Savannakhet Province according to *Plasmodium* prevalence [20] (Figure 2). In the ‘intervention’ villages, villagers were offered antimalarials and TME-supported healthcare either at an existing health facility or through a TME physician at the village. At the two ‘control’ villages, villagers were offered TME-supported health care only. Under direct observation, the ‘intervention’ villages received dihydroartemisinin piperaquine (DHAP) and a single low dose primaquine on the first day and DHAP alone was provided on the second and third days during each of the three monthly rounds of treatment. Blood samples were collected before the mass antimalarial administration and then quarterly for 12 months to detect *Plasmodium* infections [5] (Figure 2).

Under normal circumstances, villagers pay upfront for health care; during TME, residents of target village were entitled to the free routine health care, with partial financial support (up to 30% of the total cost) for major accidents and surgical conditions. Participants were also paid travel reimbursements.

## Methods

**Figure 3.**
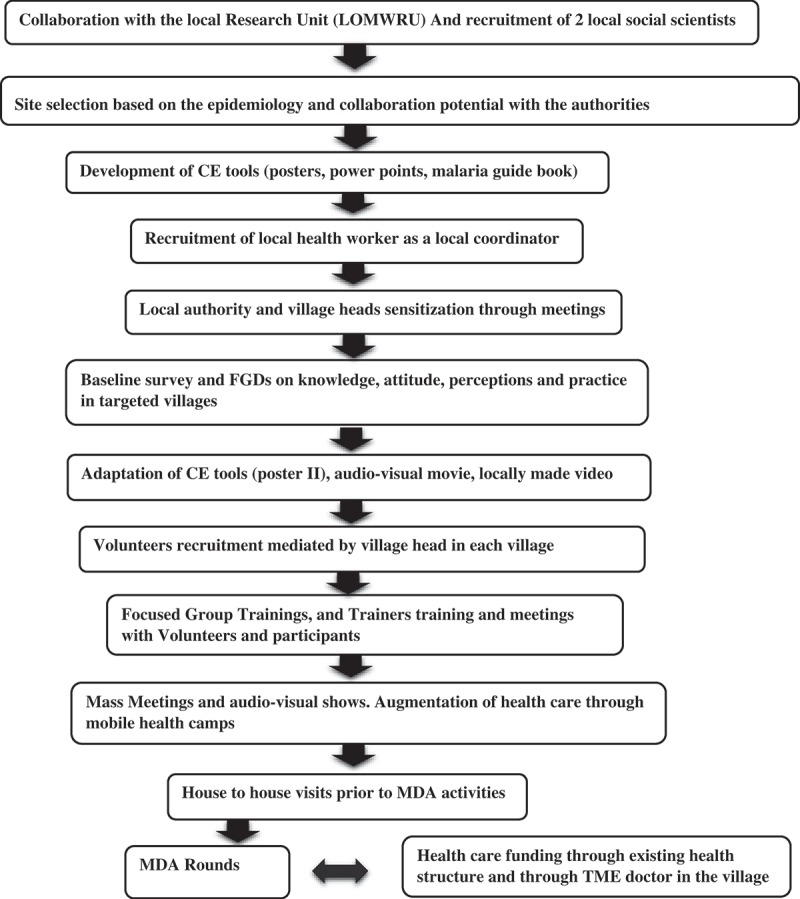
Steps in community engagement.

The data presented in this article were collected in field notes, meeting minutes and photographs taken during the process of designing and implementing community engagement for TME in Laos. Apart from early planning and joint partnership with National Malaria Control Program (Center of Malariology, Parasitology and Entomology or CMPE), community engagement began in September 2015 with initial meetings, held at provincial and district level to introduce the study to health authorities and political leaders (Figure 3). Subsequently, meetings with the heads of the TME villages were held.

Field notes (*n* = 125, roughly one field note for a community engagement activity per day, taken from September 2015 until August 2016) were based on observations made by members of the research team (BA, XS, and PK) of the activities. Field notes were taken during TME events and recorded the description of events (date, location, approximate number of participants, and theme of events), participation of villagers, informal and formal conversations, reactions and reflections by the villagers. Field staff were encouraged to reflect on their observation based on their knowledge about the significance of the events and the issues. Minutes and photographs were also taken of meetings, volunteer selections, training activities, community gatherings, house-to-house visits, and other TME activities.

Initially, published frameworks for community engagement were used to guide data analysis [16,19]. As analysis progressed it became clear that the frameworks required substantial modification to incorporate the emerging elements of the community engagement. During line-by-line coding, the codebook was therefore adapted to collate the different elements of community engagement that contributed to its success (Figure 3).

## Results

In the following section, the elements of community engagement that underpinned its success – in terms of promoting population coverage of mass antimalarial administration – are outlined. Over 85% of the target population participated in TME: in the intervention villages this entailed MDA and prevalence survey; in the control villages this involved the prevalence survey alone (Figure 2).

### Stakeholder and authority engagement

The existing partnership between Mahidol Oxford Tropical Medicine Research Unit (MORU) and Lao-Oxford-Mahosot Hospital Wellcome Trust Research Unit (LOMWRU) aided preparation for TME in Laos. LOMWRU had previously collaborated with CMPE and the Savannakhet Provincial Malaria and Parasitology Station and this became an important element of preparing for and implementing TME. Working with these partners and building on their experience of coordinating activities in the area, allowed the identification of relevant local stakeholders and with the support of the provincial malaria station, meetings with district-level leaders were organized. These meetings were intended to inform and seek their opinions concerning the objectives, design and implementation of TME. The partners also helped to identify the key person to coordinate study implementation.

Local stakeholders, including the District Head, health workers from the Nong District Health Department and health centers participated in TME and community engagement activities, such as village meetings, MDA and health care provision. The District Head’s participation in village level meetings assisted in setting up the study and promoted the authority and credibility of the project. Similarly, health workers’ involvement during MDA to provide health care further attested the authority and trust in the villages.

### Local human resources

In Vientiane, two Laotian social scientists were recruited to join the TME team (consisting of two PhD researchers, a physician, a lab assistant, a logistician and a local coordinator) and trained to design, implement and evaluate the community engagement.

The main language in the TME villages is *Lao Theung*, an oral language with no written script. To most of the TME staff, recruited from outside the villages, *Lao Theung* was incomprehensible. Only a small minority of villagers spoke *Pasha Lao*, the most widely spoken language in Laos. To deal with these language difficulties (and for other reasons outlined below) the team recruited a local coordinator who was familiar with the village authorities and spoke *Lao Theung*. Village volunteers were recruited (at first as interpreters) immediately after initial community meetings at the district health center, when TME was introduced to community leaders and prominent village members (elders, women union head and youth volunteers). The TME team, together with the village heads, interviewed candidates to select at least 10 volunteers per village who then underwent training on aspects of TME and were assigned responsibilities. Volunteers were paid per diem for their contribution to TME. Volunteers were assigned a specific number of houses within the unit (a village consisting of 80 households had around six units) for their community engagement activities, particularly reporting on and addressing villagers’ concerns in coordination with the TME team.

Village volunteers formed an integral part of TME, beginning with the planning of the TME activities, and then in coordinating and executing these activities. One notable contribution was their role in building the trust between TME and the villagers, ensuring effective two-way communication and ownership of the project.

### Formative research

The study villages had not previously been sites for biomedical research and the TME staff had little experience of remote *Lao Theung-*speaking communities. Therefore, to conduct appropriately designed community engagement, the TME team had to rapidly gain insight into the local social and economic context. In response, during the preparatory phase of the study, formative research was undertaken to identify the characteristics of the population and the general context.

This formative research was carried out prior to commencing community engagement activities in the village and continued throughout the study. Its findings influenced the overall approach, design and implementation of community engagement activities in the target communities. Data were collected through a questionnaire-based survey, focus group discussions, meetings, and observations. In addition to exploring the socio-demographic characteristics of the population, data were also collected on the knowledge, attitudes and perceptions about malaria and MDA, and the local context, such as the availability of health services, water, sanitation and hygiene and the leadership structure.

Data on socio-economic status, health care, language, knowledge, attitude and perceptions towards malaria, and MDA were helpful in adapting the community engagement activities and responding to the characteristics of particular village, particular household, and the cohorts within the sub-villages.

**Figure 4.**
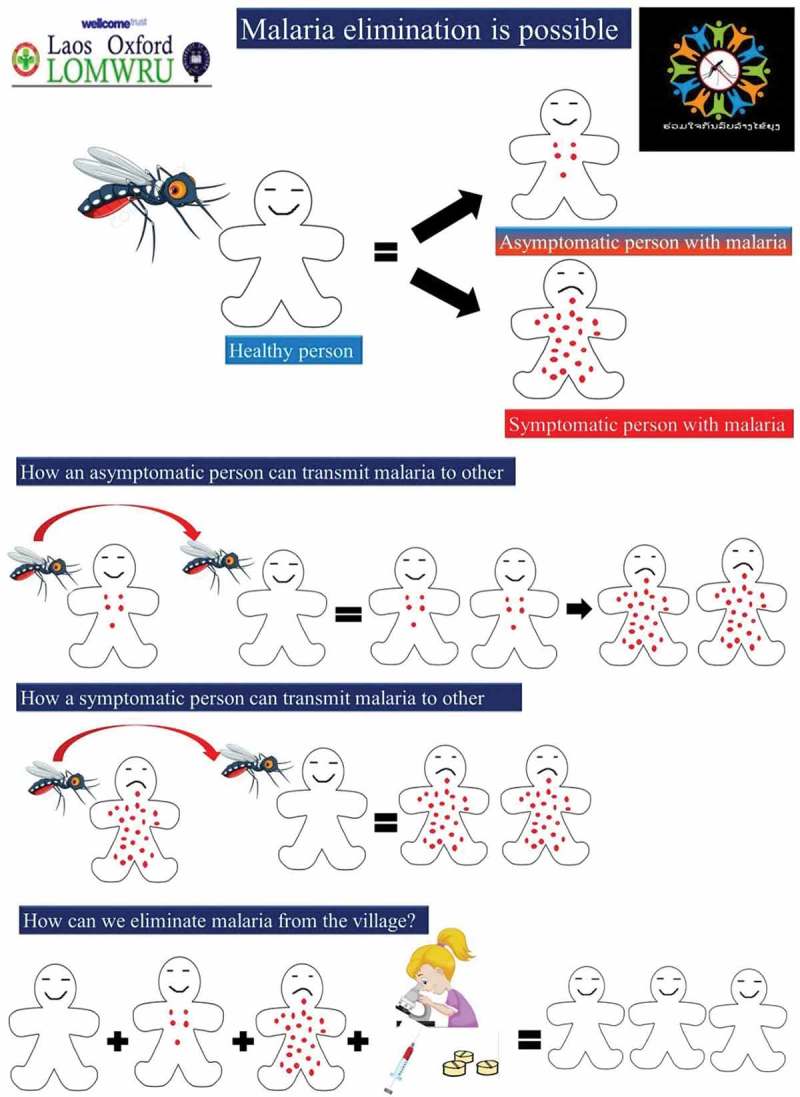
Poster I describing the symptomatic and asymptomatic malaria with rationale for MDA.

**Figure 5.**
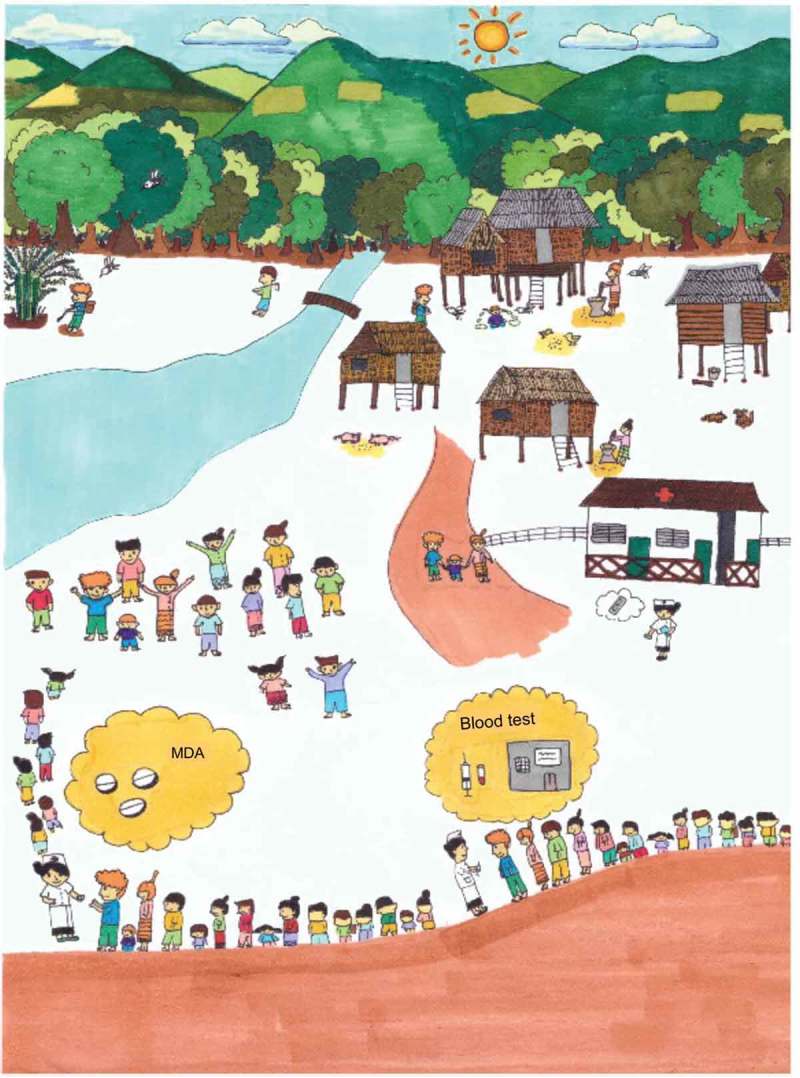
Poster II describing the contextualized picture of malaria and the elimination campaign.

The formative research, for example, strengthened the background knowledge about the low levels of literacy in the villages. For instance, poster I (Figure 4 and Appendix 1) was designed based on the previous experience in other TME sites and anticipating the low literacy level in the village, whereas poster II (Figure 5 and Appendix 1) was modified to suit the local context of the village through the findings from formative research. Poster II (Figure 5) formed part of this response and this image was also designed to approximate the local context, including a sketch that resembles life in the study villages. The research also helped to identify particular villages and the households where malaria was poorly understood or if there were rumors or misconceptions about the study. In response, volunteers and the TME team members made specific household visits and made use of health education tools.

### Responsiveness

Underpinned by the principles outlined by community engagement experts [16,19], the initial design of community engagement was based on a review of past research [11], the experience of team members at other TME sites and the findings of formative research. During the study, the community engagement strategy was altered according to the needs of the community and their responses to the various study components. These changes were informed by the ongoing monitoring that took place in the villages: the network of volunteers and TME staff made observations; meetings were held in each village with local leaders and whole communities; volunteers made house-to-house visits with the TME health education tools (posters and malaria guide book) and noted villagers’ feedback about the study and whether they had understood the health message.

Community engagement was similar across all study villages. However, because the residents of intervention villages were offered antimalarials, there were specific concerns about adverse events (both real and perceived) and large number of villagers demanded intravenous (IV) saline infusions (perceived to mitigate the weakness caused by medicine). In response to these demands, free health care was strengthened through the deployment of TME doctors in the village, there was additional health education and meetings with the affected individuals were held.

In one study village (PMM), during MDA (Figure 2), the first round (day 1) coverage was lower than in the other villagers (65%). The village volunteers therefore held meetings and garnered feedback from the community members. It became clear that there were rumors about the blood being drawn forcibly from children by restraining them in the crucifixion position, with arms stretched out. In response, study staff, along with the volunteers, visited the non-participants’ households (13 of 78 households in PMM) and provided clarifications and counselling. In subsequent days, population coverage rose notably (above 90%).

During the study, there were rumors, such as the blood draw causing people to ‘run out of blood’, ‘bringing other illnesses’, and causing ‘weakness’. Villagers were also reticent to take medicine when not sick. This resulted in part from a poor understanding of the concepts underpinning the rationale for and objectives of the study – resistance and asymptomatic malaria. In response, volunteers visited households and used the posters (Figures 4 and 5) to explain the concept of symptomatic, asymptomatic malaria and discussed the rumors about the blood draw. In village meetings, study staff also spoke about the goal of eliminating malaria from the village, asymptomatic malaria and the reasons for blood tests which were commonly misinterpreted and poorly understood by the villagers.

The formative and ongoing research highlighted the broader socio-economic and health needs of residents in the TME villages. The study was therefore able to respond to these needs and to alleviate some of the difficulties that the communities faced, for example by installing a water pump and providing completely free health care.

### Sharing control/leadership with the community

The recruitment of village volunteers by the study team with the approval of their respective village heads, promoted local ownership of the study and aimed at building trust in the villages. The volunteer training – in group meetings – aimed to ensure that they understood the rationale behind the study and its activities. In addition to providing health education, some of the village health volunteers were further trained to conduct malaria rapid diagnostic tests and were provided with medicines for first aid, including antimalarials. Volunteers provided basic health care under the guidance from TME physicians.

Through regular training and supervision by TME staff, village leaders and volunteers were involved in the day-to-day implementation of the study. Decisions on the organization of TME activities, such as the timing of the MDA, surveys, and community engagement activities were taken jointly by the village volunteers and TME staff. Their participation facilitated greater involvement of villagers, for example, through reporting their concerns about the MDA, health problems and/or adverse events linked to MDA.

## Discussion

As outlined in Box 1, the elements of community engagement that underpinned the success of TME in Laos contrast somewhat with the principles that Lavery and colleagues derived from a dengue control project in Mexico [16]. The experience of successfully implementing TME in Laos highlighted the importance of the sequence of the steps in community engagement. For instance, securing permission from the authority, an inherent component within stakeholder engagement, was the foremost step in setting up the study in Laos.

**Box 1:** Five elements of effective community engagement: lessons from a TME study in Laos.

Stakeholder and authority engagement Local human resources Formative research Responsiveness Sharing control/leadership with the community

### Stakeholder and authority engagement

Taking a stepwise approach was key to the success of community engagement for TME in Laos. This entailed sensitizing authorities, seeking consensus and collaborating with government at different levels and then selecting and training volunteers to conduct MDA. Similar processes have been reported in the community engagement that accompanied recent malaria MDAs, for example, in Vanuatu [21], Gambia [22], Nicaragua [23], Liberia [24], Cambodia [25], and Sierra Leone [26]. Although the exact nature of the individual steps and the order in which they are taken depends on the nature of the intervention and the local context, for TME in Laos, engagement with authority figures, who acted as gatekeepers was a crucial first step. The importance of taking a step-wise approach is perhaps taken for granted by researchers who have extensive experience of community engagement [16].

### Local human resources

Recent literature on community engagement has highlighted the benefits of ‘community-directed’ interventions, whereby community members take the lead in the implementation of a study or intervention, with external monitoring and supervision provided by experts [19,27,28]. Generally, this approach has two components: selecting and training villagers (building local capacity); and handing over responsibilities (increasing ownership). In recent years, building local capacity and sharing leadership to increase the ownership have been recognized as core principles in community engagement [29]. In Lao TME, volunteers were selected and trained about TME and community engagement, and assigned responsibilities. With such an approach, apart from building the local capacity, increasing the ownership and garnering villagers’ trust, Lao TME significantly benefitted from volunteers’ constant presence in the village: they acted as a conduit for villagers’ feedback, which was helpful in modifying the strategies of community engagement throughout the duration of TME. These core principles were also essential elements of successful community engagement in Vanuatu [21] and West Africa [30].

### Formative research

Formative research plays a critical role in the process of designing and implementing community engagement. Such research aims to identify potential participants’ beliefs, values, attitudes, knowledge and behaviors related to the health problem of interest and provide insight into key aspects of the local context [31,32]. With regard to malaria control programs, past failures of ‘one size fits all’ has prompted the development of a tailored approach [33]. Recently in Nigeria, such formative research was found valuable in designing the tailored chemoprevention intervention for malaria [34]. Nevertheless, in many malaria control programs, to date, little formative research has been reported in term of informing community engagement [11,33]. For Lao TME, quantitative and qualitative methods were essential to design engagement strategies for particular village and sub-villages.

### Responsiveness

Lavery and colleagues’ principles of community engagement include: ‘characterize and build knowledge of the community, its diversity, and its changing needs’ and ‘understand community perceptions and attitudes about the proposed research’ [16]. These points are certainly relevant and can be operationalized as ‘formative research’ and ‘responsiveness’. Responsiveness entails continuous monitoring of the community’s response to the intervention and the community engagement activities and adapting the approach accordingly. Providing information, which has been identified as a principle of community engagement [16], is one component of ‘responsiveness’. However, the experience of community engagement for Lao TME, suggests that it was not adequate to ‘provide information’, but rather the information must be contextually relevant and comprehensible.

### Sharing control/leadership with the community

Sharing leadership with the community is established as a goal of community engagement, whereby community members are prepared to take on responsibilities and actively participate in decision making [19,27,30]. In Lao TME, village volunteers, including members of village hierarchies were first trained, and later took on responsibilities with close supervision from TME team. Such a method of sharing the control and leadership with the community in the past has been shown to be successful when the community takes the lead and strive to achieve the goals of the project [27]. Sharing leadership with the community bears multiple benefits. An immediate benefit is the reduction of the workload. In the long term, sharing leadership increases ownership which can promote an intrinsic motivation through such as feeling of making a contribution to the community, recognition, status, knowledge & skills gained and the appreciation from stakeholders and the community [30]. For instance, in the case of Lao TME, volunteers were motivated to resolve the issues within the villages by themselves whenever they could. In Vanuatu, responsibilities were shared by community members and all the structures of the government where malaria was ultimately eliminated in addition to ongoing monitoring for new cases [21]. Similarly, in West Africa, the community took the leadership from the planning, executing and monitoring of the disease control program and achieved successful outcomes [27].

### Implications for future MDAs and health interventions

The community engagement that accompanied MDA in Lao TME was adapted to the particularities of the research site, cultural context and the study design. The study site was remote and is home to a *Lao Theung-*speaking population. In addition to their geographic and linguistic isolation, the TME villagers were research naïve and showed low levels of literacy. Nong District is one of the poorest districts (poorest in Savannakhet province) in Laos and villagers had little access to potable water and sanitation [35,36].

The site’s characteristics therefore presented challenges in terms of achieving high coverage of MDA plus regular blood draws, such as, dealing with the language barriers between the majority of study staff and villagers. This meant that communication with the villagers relied on interpreters, and therefore messages about the study and villagers’ responses to questioning were often simplified. The latter for example limited the team’s grasp of local understandings of malaria. The term ‘malaria project’ was used for TME, whether referring to the community engagement activities or the mass antimalarial administration. It was therefore difficult to separate villagers’ opinions and perceptions of the community engagement activities from the other TME activities.

Despite these challenges, Lao TME was successful in terms of securing a high participation from the community in all the activities including the achievement of high population coverage. Achieving this suggests that the community engagement was successful in spite of the adversity and that the lessons learnt from this experience can be applicable for studies and intervention programs in diverse contexts.

## Conclusion

Lao TME was successful in terms of achieving the high population coverage (above 85%) where the community engagement as an integral part of TME contributed in promotion of the uptake and the adherence of MDA. Based on the experiences of achieving high levels of coverage of MDA in a remote, linguistically isolated, resource poor and research naïve context, this article has described elements of successful community engagement. This is not intended to be a prescriptive guide for community engagement in other contexts but these elements formed the backbone of community engagement in a challenging context. Community engagement activities were partially pre-designed but undertaking formative and ongoing research was key to be able to adapt to the needs of the local communities and address emerging issues during the study.

## Figures and Tables

**Figure A1.**
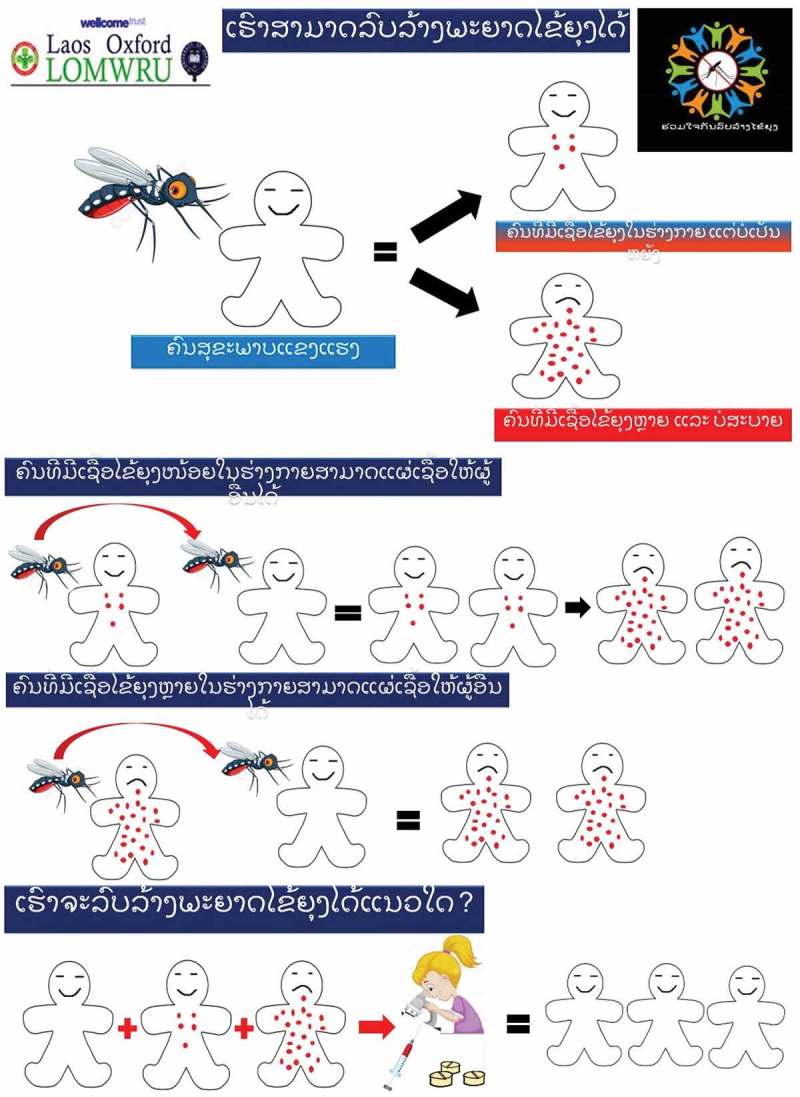
Poster I for MDA in Lao language.

**Figure A2.**
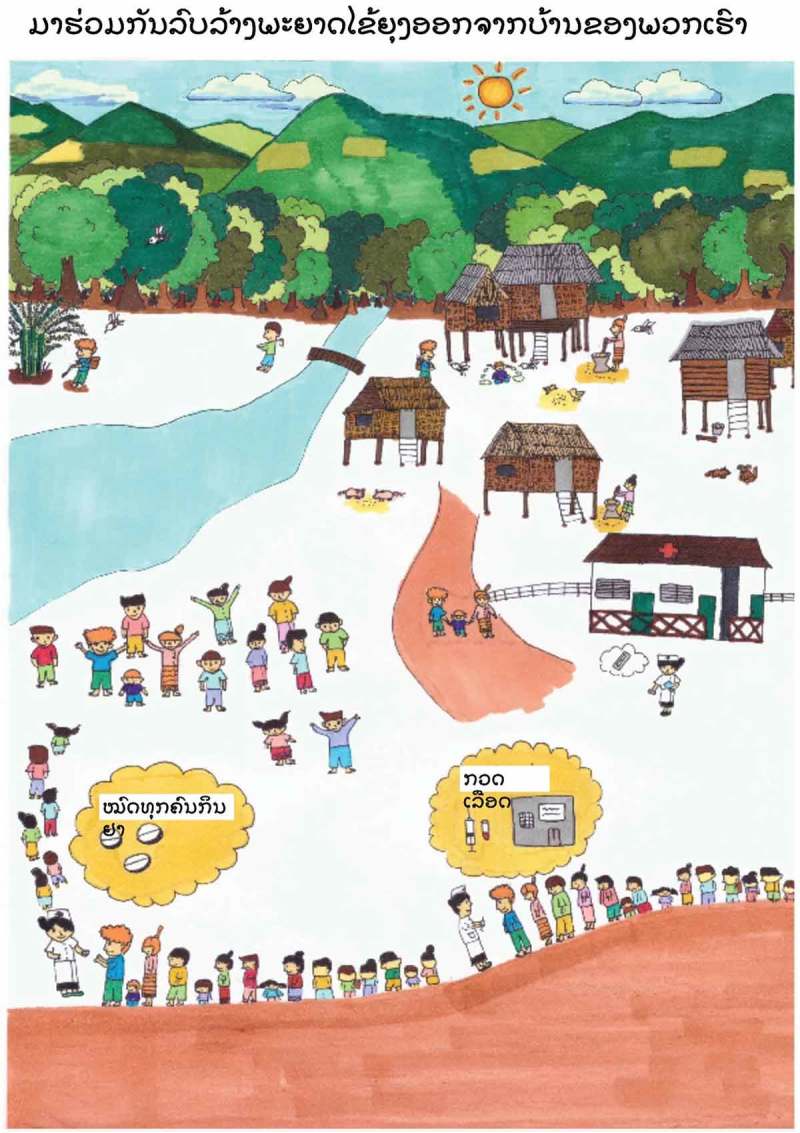
Poster II for MDA in Lao language.

## Paper context

High population coverage is key to the effectiveness of mass antimalarial administration. Community engagement activities (e.g. health education) are often used to promote coverage. Comprehensive descriptions of community engagement design and implementation are rare in the global health literature and there have been limited efforts at identifying what makes community engagement effective. We describe the community engagement for mass antimalarial administration in Laos and identify the key elements of its success.
